# *CD14* in the TLRs signaling pathway is associated with the resistance to *E*. *coli* F18 in Chinese domestic weaned piglets

**DOI:** 10.1038/srep24611

**Published:** 2016-04-21

**Authors:** Zhengchang Wu, Ying Liu, Wenhua Dong, Guo-qiang Zhu, Shenglong Wu, Wenbin Bao

**Affiliations:** 1 Key Laboratory for Animal Genetics, Breeding, Reproduction and Molecular Design of Jiangsu Province, College of Animal Science and Technology, Yangzhou University, Yangzhou 225009, P. R. China; 2 College of Veterinary Medicine, Yangzhou University, Yangzhou, Jiangsu, P. R. China

## Abstract

*Escherichia coli* F18 (*E. coli* F18) is mainly responsible for post-weaning diarrhea (PWD) in piglets. The genetic basis and regulatory mechanism of *E. coli* F18 resistance in Chinese domestic weaned piglets remain unclear. Meishan piglets were used as model animals to test their susceptibility to *E. coli* F18. By performing a comparative transcriptome study on duodenum tissues of sensitive and resistant pigs, we identified 198 differentially expressed genes (DEGs; 125 upregulated and 73 downregulated) in the resistant pigs. DEGs were predominately involved in immune system pathways, including the Toll-like receptor (TLR) signaling pathway. qPCR and western blot showed *CD14*, *IFN-α*, *TLR4* and *IL-1β*, etc. in the TLR signaling pathway had significantly higher expression levels in lipopolysaccharide (LPS)-induced small intestinal epithelial cell lines (IPEC-J2) than those in normal IPEC-J2 cells. Immunohistochemical analysis showed the increased expression of *CD14* gene in the *E. coli* F18-resistant individuals. After *CD14* knockdown, the levels of cytokines IL-6 and IL-12 were significantly reduced in IPEC-J2 cell supernatants. The adhesion ability of F18ab strain with IPEC-J2 cells was significantly increased (*p* < 0.01). This study revealed the TLR signaling pathway, and especially *CD14*, probably plays an important role in resistance to *E. coli* F18 infection in Chinese domestic piglets.

Enterotoxigenic *Escherichia coli* (ETEC) strain F18 is the main pathogenic bacterium responsible for post-weaning diarrhea (PWD) and edema disease (ED) in piglets (*Sus scrofa*)^1^,^2^. Via its fimbriae, ETEC F18 pathogen adheres to the surface of epithelial cells of the small intestines of piglets and binds to specific receptors in the brush border membrane, leading to colonization, replication, and production of enterotoxin and lipopolysaccharide (LPS)^3^. Therefore, their pathogenicity depends upon the existence of ETEC F18 receptors in the brush border membranes of small intestinal mucosal epithelial cells in piglets^4^. In previous studies on *E. coli* F18 receptors, a G/A mutation was reported at position M307 of the α-(1,2) fucosyltransferase 1 (*FUT1*) gene, which is thought to control the expression of the *E. coli* F18 receptor. *FUT1* was proposed as a candidate gene for the selective breeding of *E. coli* F18 adhesion-resistant pigs. At the mutation site in *FUT1*, G is dominant to A; i.e., pigs with the *AA* genotype show resistance to *E. coli* F18, whereas pigs with the *GG* or *AG* genotypes are sensitive to *E. coli* F18^5^,^6^. Recently, the *AA* genotype at M307 has been detected in Western commercial pig breeds, including Duroc, Pietrain, Yorkshire and Landrace; however, animals with this genotype have not been found in native Chinese pigs^7^,^8^, which makes breeding of resistant native Chinese pig breeds difficult. Previously, we established Sutai pig (a new hybrid between the Duroc and Meishan breeds) populations that are resistant and sensitive to *E. coli* F18. Using Agilent two-color microarray-based gene expression profile, we analyzed the differential gene expression and important pathways in Sutai pigs resistant and sensitive to *E. coli* F18 (data submitted to Gene Expression Omnibus, Accession number GSE26854)^9^. This analysis identified the glycosphingolipid biosynthesis-globo series pathway, including *FUT1, FUT2, ST3GAL1, HEXA, HEXB, B3GALNT1* and *NAGA* genes. Nevertheless, Sutai pig with foreign Duroc DNA cannot completely reveal the regulatory mechanism of *E. coli* F18 resistance in native Chinese pig breeds. In addition, Zhou *et al.* analyzed the differential gene expression profile in intestinal epithelial cell with ETEC infection; however, they only analyzed the mechanism of bacterial infection at a cellular level^10^. The lack of verification of symptom change of piglets’ phenotype meant that effective screening and identification of resistant and sensitive individuals to *E. coli* F18 has become bottleneck for exploring the genetic basis of *E. coli* F18 resistance.

Recently, with the development of next-generation sequencing (NGS) technologies, RNA sequencing (RNA-seq) has rapidly emerged as a major transcriptome profiling system. With the help of adapted bioinformatic tools, RNA-seq allows the exploration of the transcriptome in an unprecedented manner in terms of accuracy and data insight^11^. Meanwhile RNA-Seq technology can not only detect variations in gene expression patterns, but also identify splicing events, new isoforms, and different promoter and polyadenylation signal usage. However, it is difficult to analyze the molecular mechanism of *E. coli* F18 resistance using RNA-Seq technology in native Chinese pig breeds because of the lack of extreme phenotype individuals for *E. coli* F18 infection.

One of main purposes of this study was to obtain *E. coli* F18-resistant and -sensitive individuals in native Chinese pig breeds. In this study, Meishan piglets were used as model animals to test their susceptibility to *E. coli* F18 by challenging them through feeding them with *E. coli* F18 strains. Using a series of tests, such as *E. coli* F18 bacteria detection, bacteria counting and adhesion test of the pathogens to the epithelial cells of small intestine *in vitro*, we identified *E. coli* F18-resistant and -susceptible complete sib-pair individuals. In pigs, the functions of the small intestine include the establishment of a physical and immunological barrier against foreign antigens, such as natural toxins, and pathogenic and commensal microorganisms^12^. *E. coli* F18 strain colonizes and replicates mainly in the duodenum. Based on the identified *E. coli* F18-resistant and sensitive individuals, to obtain detailed genetic information about the resistance mechanism, we sequenced the duodenal transcriptomes of Meishan piglets resistant and sensitive to *E. coli* F18 using a high-throughput sequencing platform – Illumina HiSeq 2000. We obtained many differentially expressed genes (DEGs) between the resistant group and sensitive group, which were analyzed by Gene Ontology (GO) and pathway enrichment analysis to identify important pathway or functional genes related to *E. coli* F18 infection. Furthermore, we used quantitative real-time PCR (qRT-PCR) technology to detect the expression of important pathway genes in small intestinal epithelial cell line (IPEC-J2) under LPS treatment. Additionally, an important gene was identified by gain- and loss-of-function assays in IPEC-J2 cells. This study determined the molecular regulation mechanisms and the critical genes involved in regulating piglets’ responses to *E. coli* F18 infection, which will pave the way for further genetic engineering to improve resistance to *E. coli* F18 in Chinese native pig breeds.

## Materials and Methods

### Ethics statement

The Institutional Animal Care and Use Committee (IACUC) of the Yangzhou University Animal Experiments Ethics Committee approved the animal study proposal, with the permit number: SYXK(Su) IACUC 2012-0029. All piglet experimental procedures were performed in accordance with the Regulations for the Administration of Affairs Concerning Experimental Animals approved by the State Council of the People’s Republic of China.

### Challenge with *E. coli* F18 strain and tissue sample collection

Meishan weaning piglets were collected from Kunshan Conservation Ltd. (Suzhou City, Jiangsu Province, China). We selected three litters of weaning piglets at 35 days of age, 12 piglets per litter, with almost same birth weight and weaning weight. The challenge experiment with *E. coli* F18 strain was carried out according to previously described methods^13^. We collected feces from piglets in the same litter weaned after 30 days in the same feeding environment, which were used to detect common pathogens, including general pathogenic *Escherichia coli* (*E. coli* F18, *E. coli* K88) and viruses (transmissible gastroenteritis virus (TGEV), porcine epidemic diarrhea virus (PEDV), rotavirus (RV)) (PCR primers are shown in Supplementary Table S1). Twelve piglets per litter were randomly divided into two groups: the control group (two piglets) and the experimental group (ten piglets). Each piglet was housed individually in separate pens. They were fed ad libitum with a commercial-type compound feed for weaned piglets containing 21.7% crude protein, without antimicrobial additives and organic acids. Beginning at day 3 post-weaning, experimental piglets were challenged with a daily dose of 4.6 × 10^8^ CFU of *E. coli* F18 strain once a day for up to 10 days, or until they showed diarrhea. No additional food was given and we ensured that piglets ate all food before the challenge experiment. Throughout the experiment, fecal shedding of the inoculated bacteria was monitored by daily fecal sampling and feces consistency was scored using the parameters “normal”, “pasty” and “watery”. Only piglets with watery feces were considered as diarrheic. The intestinal tracts of the diarrheic pigs were used to carry out a series of experiments, such as *E. coli* F18 bacteria counting, histopathological detection and adhesion test of the pathogens to the epithelial cells of small intestine *in vitro*^14^,^15^. The animals were allowed access to feed and water ad libitum under normal conditions and were humanely sacrificed using an intravenous injection of pentobarbital sodium as necessary to ameliorate suffering. About 100 mg of duodenal tissue was removed and the scraped epithelium of the duodenum was placed into 1.5 ml nuclease-free Eppendorf tubes, frozen in liquid nitrogen and stored at −80 °C until further use.

### RNA isolation, cDNA library preparation and sequencing

Total RNA was isolated using the Trizol Reagent (Invitrogen Life Technologies), after which the concentration, quality and integrity were determined using a NanoDrop spectrophotometer (Thermo Scientific) and a Bioanalyzer 2100 system (Agilent). Finally, six samples with RNA integrity number (RIN) values above 8 were used for libraries construction. Three micrograms of RNA were used as input material for the RNA sample preparations. Sequencing libraries were generated using the TruSeq RNA Sample Preparation Kit (Illumina, San Diego, CA, USA). Briefly, mRNA was purified from total RNA using poly-T oligo-attached magnetic beads. Fragmentation was carried out using divalent cations under elevated temperature in an Illumina proprietary fragmentation buffer. First strand cDNA was synthesized using random oligonucleotides and SuperScript II. Second strand cDNA synthesis was subsequently performed using DNA Polymerase I and RNase H. Remaining overhangs were converted into blunt ends via exonuclease/polymerase activities and the enzymes were removed. After adenylation of the 3′ ends of the DNA fragments, Illumina PE adapter oligonucleotides were ligated to prepare for hybridization. To select cDNA fragments of the preferred 200 bp in length, the library fragments were purified using the AMPure XP system (Beckman Coulter, Beverly, CA, USA). DNA fragments with ligated adaptor molecules on both ends were selectively enriched using Illumina PCR Primer Cocktail in a 15 cycle PCR reaction. Products were purified (AMPure XP system) and quantified using the Agilent high sensitivity DNA assay on a Bioanalyzer 2100 system (Agilent). The sequencing library was then sequenced on a Hiseq 2000 platform (Illumina) by Shanghai Personal Biotechnology Cp. Ltd.

### Sequence reads mapping and assembly

Raw data (raw reads) in the fastq format were first processed using in-house perl scripts. In this step, clean data (clean reads) were obtained by removing reads containing adapters, reads containing poly-N and low quality reads from the raw data. At the same time, Q20 (the amount of bases with an error rate ≤1%), Q30 (the amount of bases with an error rate ≤0.1%), GC content and sequence duplication level of the clean data were calculated. All the downstream analyses were based on the high quality clean data. The reference genome and gene model annotation files were downloaded from the *Sus scrofa* reference genome (Sscrofa v10.2 sequence)^16^. An index of the reference genome was built using Bowtie v2.0.6^17^ and paired-end clean reads were aligned to the reference genome using TopHat v2.0.4^18^. We selected TopHat as the mapping tool because TopHat can generate a database of splice junctions based on the gene model annotation file and thus a better mapping result is produced compared with other non-splice mapping tools. Clean reads were aligned to the reference genome through SOAP2^19^, and then duplicated reads and multiply-mapped reads were filtered from the alignment results to eliminate the PCR interference and ambiguous mapping. The clean reads are available at NCBI’s SRA repository (PRJNA271310 BioProject; BioSample accessions: SAMN03273387, SAMN03273895, SAMN03273896, SAMN03273897, SAMN03273910 and SAMN03273911).

### Gene annotation and functional analysis

In this study, EggNOG (evolutionary genealogy of genes: Non-supervised Orthologous Groups; http://eggnog.embl.de/version_3.0/), a database of orthologous groups of genes, was used to annotate genes with common denominators or functional categories. GO enrichment analysis of differentially expressed genes was implemented by the GOseq R package^20^, in which gene length bias was corrected. GO terms with corrected *P* value less than 0.05 were considered significantly enriched by DEGs. We used the KOBAS software^21^ to test the statistical enrichment of DEGs in Kyoto encyclopedia of genes and genomes (KEGG) pathways.

### Differential expression analysis of transcripts

HTSeq v0.5.3 (http://www-huber.embl.de/users/anders/HTSeq) was used to count the number of reads mapped to each transcript. The reads per kilobase of transcript sequence per million mapped reads (RPKM) value for each transcript was measured^21^. The RPKM was calculated based on the mapped transcript fragments, transcript length and sequencing depth. This is a commonly used method for estimating transcript expression. Cuffdiff 22 and DESeq^23^ methods identified the DEGs between the different groups. For the DESeq method, DEGs were detected using the Deseq R package (1.8.3). We first mapped high-quality reads to unigenes to calculate the number of reads mapped to each unigene in six samples. These raw read counts were then used as the input for DESeq to obtain the normalized signal for each unigene, and the fold change of unigene expression values, with *p*-values, were compared to each other among the six samples used to report the differential expression. A corrected *p*-value of 0.05 was set as the threshold for significant differential expression.

## Validation of differentially expressed genes and signaling pathway genes by quantitative real-time PCR

### Primer design

To validate the repeatability and reproducibility of gene expression data obtained by RNA sequencing in *Sus scrofa*, several genes of interest were validated by using quantitative real-time PCR (for the primers, see Supplementary Table S2). The expressions of TLR4-mediated Toll-like receptor (TLR) signaling pathway genes, including *TLR4*, *CD14*, *MyD88*, *TNF-α*, *IL-1β* and *IFN-α* were detected in pig epithelial cell line (IPEC-J2) under LPS treatment (for the primers, see Supplementary Table S3). All Primers were designed according to Illumina sequencing data using Primer 3 (http://fokker.wi.mit.edu/primer3/input.htm), and were synthesized by Sangon Biotech (Shanghai, China).

### Cell culture and LPS treatment

The pig epithelial cell line IPEC-J2 (contributed by the University of Pennsylvania, Philadelphia, PA, USA) was cultured in complete culture solution (Dulbecco’s modified Eagle medium, DMEM:F12 medium = 1:1, 10% fetal calf serum; Gibco BRL, Life Technologies, Grand Island, NY, USA). When the cells were 80–90% confluent, they were exposed to 0.1 μg ml^−1^ LPS and 1 μg ml^−1^ LPS (Sigma-Aldrich, St. Louis, MO, USA). The negative control was incubated with the cell culture solution used for preparing the LPS; three parallel replicates were used for each group. Cellular RNA was extracted after 2, 4, and 6 h, while negative control RNA was collected after 6 h.

### Quantitative real-time PCR (qPCR)

Total RNA was extracted using the TRIzol Reagent (Takara), according to the manufacturer’s instructions. The housekeeping genes *ACTB* and *GAPDH* were used as a reference control to normalize the expression level. We performed qPCR using an ABI 7500 system (Applied Biosystems, Foster City, CA, USA). Each reaction volume contained 10 μl 2× SYBR Premix Ex *Taq* II (Takara, Dalian, China), 0.4 μl forward primer (10 μM), 0.4 μl reverse primer (10 μM), 0.4 μl 50× ROX Reference Dye II (Takara, Dalian, China), and 2 μl cDNA, and ddH_2_O to 20 μl. The amplification program was as follows: initial denaturation at 95 °C for 30 s; followed by 40 cycles of denaturation at 95 °C for 5 s and annealing at 60 °C for 34 s. To analyze the specificity of the amplified products, we collected multiple information points for melting curve analysis. The program was as follows: 95 °C for 15 s, 60 °C for 1 min; and 95 °C for 15 s, 60 °C for 15 s. The comparative CT method (2^−∆∆CT^ method)^24^ was used to analyze the expression level of different genes.

### Western blot analysis

To confirm the role of the signaling pathway identified in *E. coli* F18-resistent and -susceptible piglets, total proteins were extracted with a NE-PER kit (Thermo Fisher Scientific) according to the manufacturer’s protocol. Protein levels were normalized using a BCA kit (Thermo Fisher Scientific). SDS-PAGE conditions were, 10 μl protein loaded to a 10% gel run at 120 V for 90 min. For western blotting, proteins were transferred to PVDF membranes and immunoblotted with primary antibodies against NFkB (1:1000), CD14 (1:800), ERK (1:1000), IFNγ (1:600), IL-1β (1:600), JNK (1:1000), p38 (1:1000), TNFα (1:600), β-actin (1:4000) (Abcam, Cambridge, MA, USA). The secondary antibody was horseradish peroxidase (HRP) conjugated goat anti-rabbit IgG (Jackson ImmunoResearch Laboratories, West Grove, PA, USA., 1:5000).

### Immunohistochemical analysis of *CD14* gene

Duodenal tissues were collected from *E. coli* F18-resistent and -susceptible individuals. Serial sections of paraffin-embedded tissues were first treated with 3% peroxide for 15 min to quench endogenous peroxidase. The sections were rinsed for 15 min with 0.01M PBS (Jiangsu KeyGEN BioTECH Corp., Ltd), and then incubated at room temperature for 2 h with the following primary antibodies: rabbit anti-CD14 (Abcam, Cambridge, MA, USA, 1:100). Whereafter, they been sequentially incubated with the HRP conjugated secondary antibody (Jackson ImmunoResearch Laboratories, West Grove, PA, USA., 1:50) for 30 min at room temperature with diaminobenzidine (DAB) as the substrate chromogen (Jiangsu KeyGEN BioTECH Corp., Ltd), the sections were counterstained with hematoxylin.

## RNA interference of *CD14* gene and functional analysis

### Lentivirus shRNA knockdown of *CD14*

Four RNAi sequences targeting *CD14* mRNA (#1: 5′-GCCAAGCCTCAAGGTACTGAA-3′; #2: 5′-ACCCTGGACCTATCTGACAAT-3′; #3: 5′-GCTCTCTCAATTTGTCATTCG-3′; #4: 5′-GCTGCAACAAGCTGAACAGAG-3′) and one off-target control sequence (#NC: 5′-TTCTCCGAACGTGTCACGTTTC-3′) were cloned separately into LV3-H1/GFP&Puro vector (GenePharma) and cotransfected into 293T cells (GenePharma) together with packaging plasmids. The virus was collected and used to infect the target cells (IPEC-J2). After 48 h of incubation, transfected cells were selected by blasticidin (1 μg ml^−1^, InvivoGen). The cells infected with #1, #2, #3, #4 lentivirus *CD14* shRNA vector were designated as Lenti-RCD14-n (n = 1, 2, 3, 4) cells, respectively.

### RNA isolation and qRT-PCR

Total RNA was isolated from IPEC-J2 control and Lenti-RCD14-n cells using the TRIzol Reagent and was reverse transcribed into cDNA using PrimerScript RT Master Mix (Takara). Real-time RT-PCR was performed using the SYBR premix ExTaq (Takara, Dalian, China). The relative changes in gene expression were analyzed using the 2^−ΔΔCt^ method^24^.

### Cytokines detection in the TLR signaling pathway and *E. coli* F18 adhesion *in vitro*

In IPEC-J2 control and Lenti-RCD14-n cells, we measured five cytokines in the TLR signaling pathway including interleukin-6 (IL-6), interleukin-8 (IL-8), interleukin-12 (IL-12), macrophage inflammatory protein-1α (MIP-1α), macrophage inflammatory protein-1β (MIP-1β) using the bio-plex 100 system (BIO-RAD Laboratories, Hercules, CA, USA) with Procarta Immunoassay Kits (Affymetrix, CA, USA) according to the manufacturer’s instructions.

The F18ab fimbriae standard strain 107/86 (O139:K12:H1) and F18ac (8199) were provided as a gift by the veterinary laboratory at the Institute of Microbiology, University of Pennsylvania. For IPEC-J2 control and Lenti-RCD14-n cells, F18 fimbriae adhesion *in vitro* was performed according to the method of Liu *et al.*^25^. To establish an efficient and accurate method to detect the adhesion ability of *E. coli* to small intestinal epithelial cells in pigs, a relative quantification method was used. Using Primer Express 2.0 software, we designed primers for the gene encoding the *E. coli* fimbria protein, *PILIN* (GenBank NO. M25302.1): F: 5′-AGGCCGAACCAAAGAAGCAT-3′, R: 5′-TCACCATCAGGGTTTCTGAGT-3′; and for the housekeeping gene *β-ACTIN* (GenBank NO. NC_010445.3): F: 5′-GTCGTACTCCTGCTTGCTGAT-3′, R: 5′-CCTTCTCCTTCCAGATCATCGC-3′. We took mixed DNA of cells and bacteria as templates for triplicate reactions, and the relative quantification method was used to detect DNA amplification.

## Results

### Identification of Meishan piglets sensitive and resistant to *E. coli* F18

**Figure 1:**
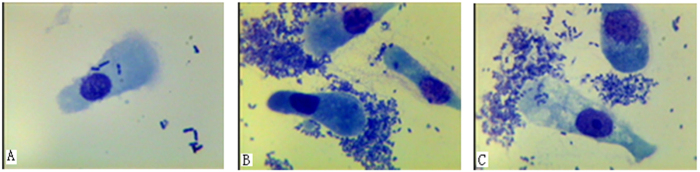
Adhesion test for intestinal epithelial cells for *E. coli* F18-resistant and -sensitive piglets. The adhesion of *Escherichia coli* F18 to intestinal epithelial cells in Meishan piglets, (**A**) represents F18-resistant piglets displaying no adherence with F18-expressing fimbriae of the standard ETEC strain; (**B**) represents F18ab-susceptible piglets displaying a large amount of adherence with F18ab-expressing fimbriae of the standard ETEC strain, (**C**) represents F18ac-susceptible piglets displaying a large amount of adherence with F18ac-expressing fimbriae of the standard ETEC strain. Photos were taken with an oil immersion lens at 1000× magnification.

Before the bacterial challenge experiment, we used PCR technology to detect common pathogens, including transmissible gastroenteritis virus (TGEV), porcine epidemic diarrhea virus (PEDV), rotavirus (RV), *E. coli* F18 and *E. coli* K88. PCR products of the above pathogens were not detected in the piglets’ feces (Supplementary Fig. S1), which indicated experimental piglets were not infected by common pathogens. During the challenge experiment with *E. coli* strain F18, two extreme phenotypes, “watery diarrhea” and “normal”, appeared in experimental group. Nine piglets showing “watery diarrhea” were defined as the “diarrhea group” and eight normal piglets as the “normal group”. After slaughter, we took intestinal tissues (duodenum, jejunum and ileum) to detect bacterial numbers. The number of bacteria in the intestinal contents of the diarrhea group (average CFU: 7.0 × 10^8^ ~2.0 × 10^10^ ml) was significantly more than that of normal piglets (average CFU: 1.8 × 10^7^ ~ 1.0 × 10^8^ ml) (Supplementary Table S4). Moreover, the result of pathological tissue section showed that the villus height of pathological intestinal mucosa was shorter, the congestion phenomenon existed in the clearance of ileum tissue, and the villus of the ileum mucous layer fell off (Supplementary Fig. S2). In all piglets detected by the binding assay, we strictly identified piglets displaying no adherence with F18-expressing fimbriae of the standard *ETEC* strain as *E. coli* F18-resistant individuals (Fig. 1A). In contrast, piglets displaying a large amount of adherence were identified as *E. coli* F18-susceptible individuals (Fig. 1B,C). Analysis of the observed extreme phenotypes and the experimental verification obtained three complete sib pairs of *E. coli* F18-susceptible and *E. coli* F18-resistant individuals for transcriptome analysis.

### Transcriptome analysis of swine duodenum tissue

The duodenum tissue transcriptome was sequenced from six piglets (Resistant, R = 3, Sensitive, S = 3) with extreme phenotypes for *E. coli* F18 infection. We obtained approximately 40.97, 47.25, 31.64, 39.58, 48.81, and 36.41 million raw reads for R1, R2, R3, S1, S2 and S3, respectively. 244.6 Mb of 100 bp paired-end reads was acquired from the RNASeq experiment (Supplementary Table S5). Sequence alignment was performed against the reference pig genome (Sscrofa10.2) using Tophat^18^. After quality control, about 80.1% (76.9–82.1%) of reads were mapped to the reference genome. Of them, 91.5–91.8% mapped uniquely to the pig reference genome, and 8.2–8.6% showed multiple matches (hereafter, the matches are referred to as unique and multiple matches) (Supplementary Table S6). The genomic distribution patterns of uniquely mapped reads showed that a majority of the reads were located in annotated genic regions, with an average of 85.2% (83.9–86.1%) of the mapped reads corresponding to annotated genes, and 93.3% (90.4–95.4%) of them were located in exons. The remaining 14.7% (13.9–16.1%) of reads mapped to intergenic regions, indicating that they were not annotated in the reference genome (Supplementary Table S7).

The gene-expression distribution was similar in both groups, classifying 29% of the selected genes between 0–20 mapped reads; 16% among 20–200 mapped reads; 30% among 200–2,000 mapped reads; 21–22% among 2,000–20,000 mapped reads, and the remaining genes (1.8–1.9%) with more than 20,000 mapped reads (Supplementary Fig. S3). The correlation of transcripts expression between samples is the most important indicator for the reliability of experimental results and the rationality of sampling. Generally, the correlation value should be up to 0.92 (R^2^ ≥ 0.92). The correlation coefficient (R^2^) between the two individuals within the resistant and sensitive groups for *E. coli* F18 infection was 0.92 to 0.98, indicating that the similarity of the three biological replicates within each group was sufficiently high (Supplementary Fig. S4). We further examined the quality of our transcriptome library and the effectiveness of the annotated process. A total of 717 unigenes with EggNOG classification were grouped into 20 EggNOG categories, including “RNA processing and modification”, “Cell motility”, “Extracellular structures”, “Undetermined” and “Nuclear structure” having no annotated unigenes (Supplementary Fig. S5). In this study, there were four different splice patterns detected in porcine duodenum transcriptome data, which included alternative 5′ splicing site (A5SS), alternative 3′ splicing site (A3SS), Exon skipping (ES) and intron retained (IR). Of these, ES, IR and A5SS were the major splicing patterns detected, which represented 85.8% of the total splicing events (Supplementary Fig. S6).

### Differentially expressed genes involved in resistance and sensitivity to *E. coli* F18

To better survey the biological mechanism regulating resistance to *E. coli* F18 infection, we identified the DEGs between the two groups (S & R). Supplementary Fig. S7 shows that, at the selected cut-off (−log10(*p*-value) >1.7 or p-value < 0.05), there was a clear departure from the expected result among transcripts accepted as differentially expressed (indicated by the blue trend being above the strait red line). 198 DEGs were detected between the two groups when | fold changes |≥2 and *p* < 0.05 were used as cutoff values. Of these, 125 DE genes were upregulated, while 73 DE genes were downregulated in the *E. coli* F18-resistant group compared with the susceptible group (Supplementary Fig. S8, Table S8).

### Gene Ontology (GO) and Pathway functional enrichment analysis of differentially expressed genes

To annotate the duodenum transcriptome, GO terms were assigned to porcine unigenes based on their identity to known protein sequences in the *Sus Scrofa* database and nr database. 198 DEGs were classified into 103 functional groups according to the GO project for biological processes. The top 12 biological process groups of the genes are shown in Supplementary Fig. S9. Upregulated and downregulated genes were both involved in “immune system process”, (3.9%). Further GO enrichment analysis of all DEGs (Supplementary Fig. S10) showed that “immune system process” had the most significant enrichment degree in the biological process groups, where the *CXCL11*, *OPN*, *CD14* and *MMP7* genes were significantly enriched.

**Figure 2:**
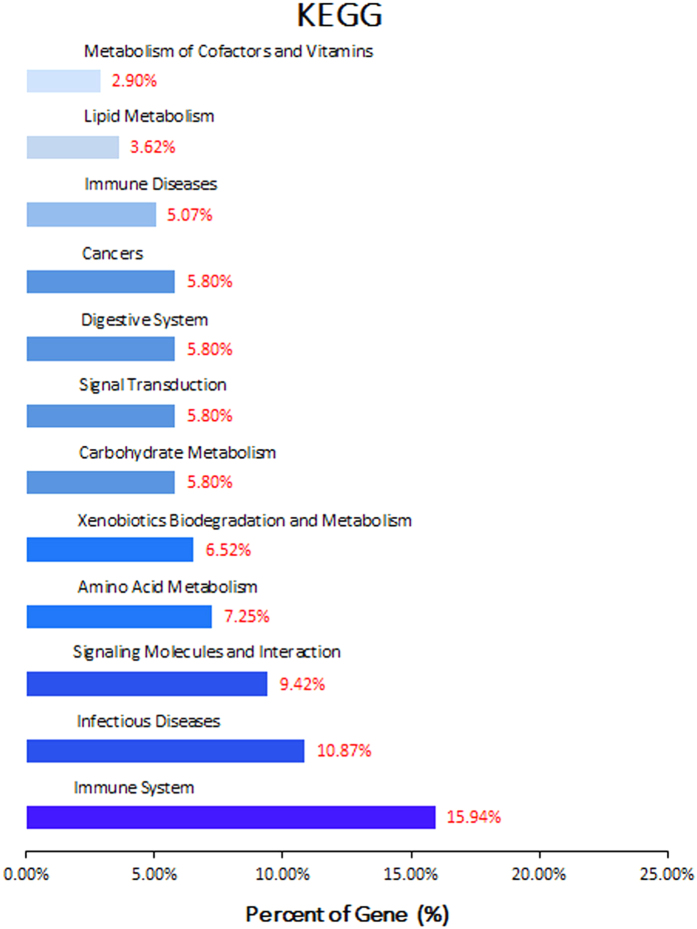
KEGG pathway analysis for differentially expressed genes. The significant pathways for differentially expressed genes. A *p*-value < 0.05 was used as a threshold to select significant KEGG pathways.

To further define the functions of DEGs in duodenum tissue after *E. coli* F18 infection, the KEGG database was used to analyze pathways. The results showed that the DEGs were mainly involved in the “Immune System” pathway (15.94%) and “Infectious Diseases” pathway (10.87%) (Fig. 2). According to enrichment degree, we identified an interesting pathway in the “Immune System” pathways, namely TLR4-mediated Toll-like receptor signaling pathway, where *CD14*, *OPN*, *CXCL11* genes were significantly enriched. Moreover, in the “Infectious Diseases” pathways, the *CD14* gene was significantly enriched in both the pathogenic *Escherichia coli* infection pathway and the Salmonella infection pathway.

### Validation of differential expressed genes of RNA-Seq analysis by qPCR

**Figure 3:**
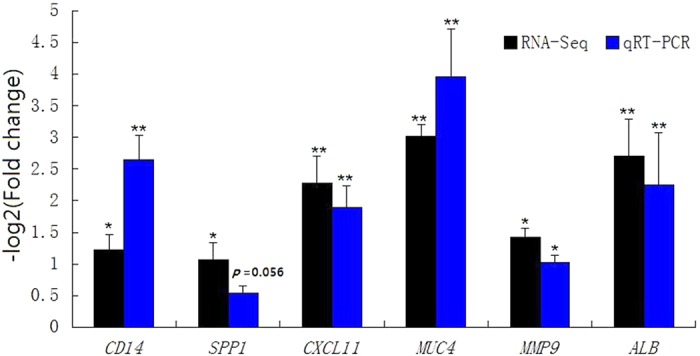
Validation of the RNA-Seq expression profiles of selected DEGs by qRT-PCR. Fold change means *E. coli* F18-susceptible group/*E. coli* F18-resistant group. *p < 0.05; **p < 0.01.

Real-time PCR was used to validate selected differentially expressed genes identified from the RNA-seq data. Six differentially expressed genes (*CD14, MUC4, SPP1, MMP9, CXCL11* and *ALB*) were selected from the DEGs, all of which were upregulated between the two groups. The real-time PCR results revealed the similar relative pattern of transcription as the RNA-Seq data, thereby validating and confirming the RNA-Seq results, indicating that the expression levels of these genes were significantly upregulated in response to *E. coli* F18 infection (Fig. 3).

### Expression of TLR4-mediated Toll-like receptor signaling pathway genes in IPEC-J2 cells following LPS exposure

**Figure 4:**
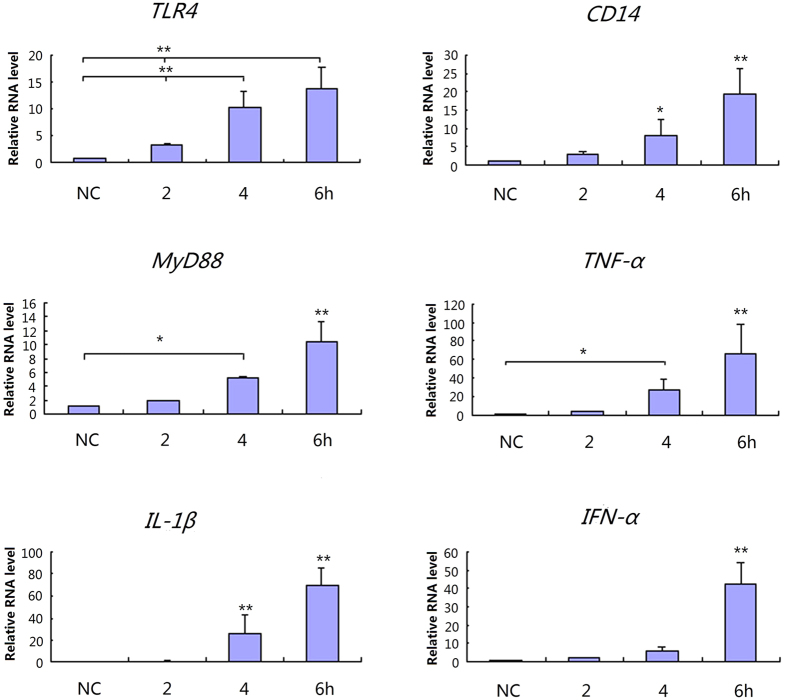
Gene expressions of the TLR4 signaling pathway in IPEC-J2 induced by 0.1 μg mL^−1^ LPS. NC represents negative control, *p < 0.05; **p < 0.01.

The expression quantities of each gene in the negative control were set as 1, and we used the results of fluorescence quantitation to represent the expression levels. In cells exposed to 0.1 μg mL^−1^ LPS, the expression quantities of each gene increased, with a stepped distribution (Fig. 4). The expression of all genes increased at 2 h, but the differences were not significant. After 4 h, the expressions of all but *IFN-α* were significantly higher than in the negative control. *CD14*, *TLR4*, and *IL-1β* expression was remarkably higher at 4 h than at 2 h (*p* < 0.01). At 6 h, the expressions of all but *TLR4* were significantly higher than the negative control and the values at 2 h and 4 h.

**Figure 5:**
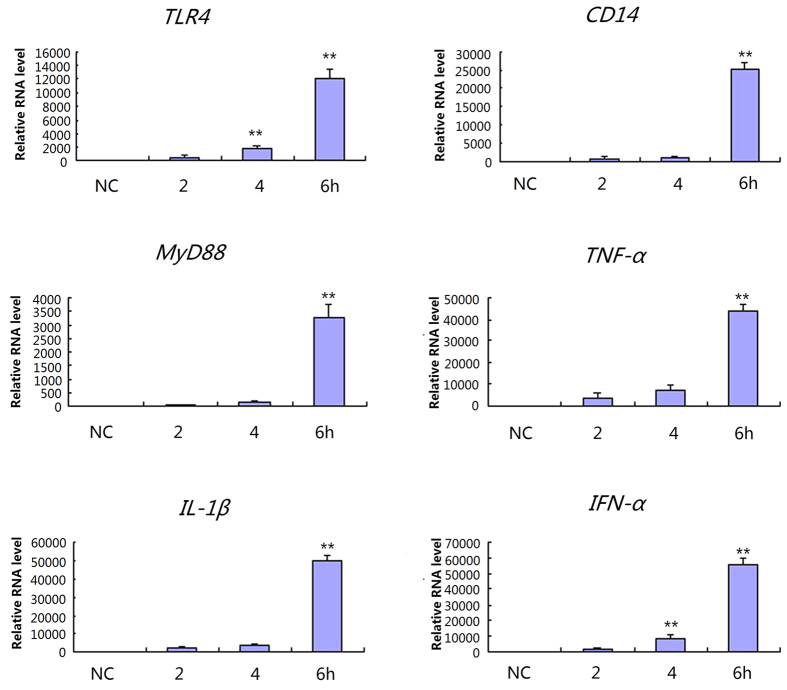
Gene expressions of the TLR4 signaling pathway in IPEC-J2 induced by 1 μg mL^−1^ LPS. NC represents negative control, *p < 0.05; **p < 0.01.

**Figure 6:**
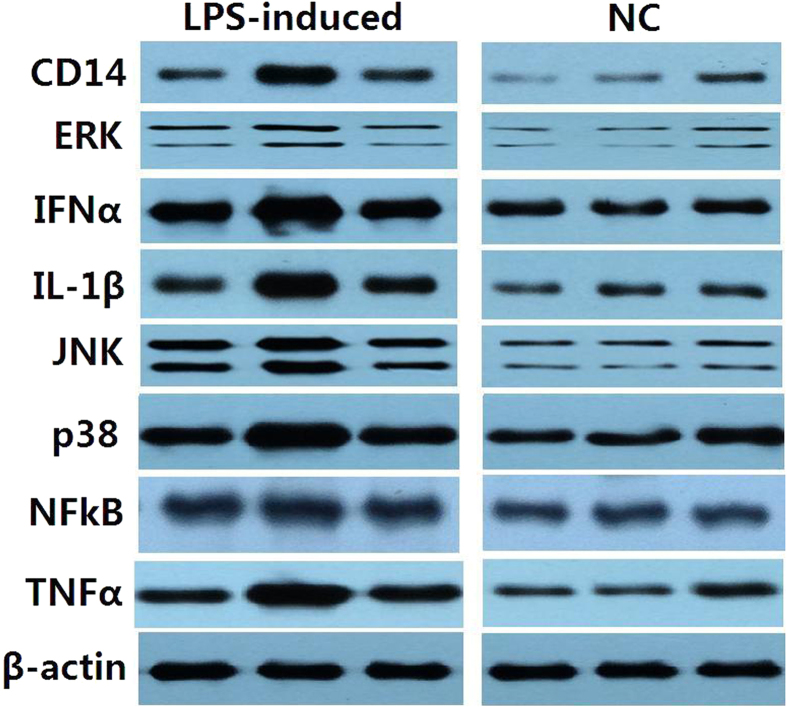
Differential expression of TLR4 signaling pathway proteins between normal and LPS-induced IPEC-J2 cells, analyzed by western blot. Each image shows three samples from cells. NC represents negative control.

There was a sharp increase in the expression of all genes in cells exposed to 1 μg mL^−1^ LPS at 4–6 h (Fig. 5). Moreover, *TLR4* and *IFN-α* expression after 4 h was remarkably higher than the negative control and that at 2 h (*p* < 0.01). The expressions of all genes in the test pathway were significantly different from that of other groups after 6-h of exposure to LPS (*p* < 0.01). Further western blot analysis showed the expression of TLR signaling pathway proteins such as NFκB, ERK, p38, JNK, IL-1β, TNFα, IFN-α and CD14 were up-regulated in IPEC-J2 cells following LPS exposure (Fig. 6).

### *CD14* immunohistochemical staining, RNA interference and its relationship with resistance to *E. coli* F18

**Figure 7:**
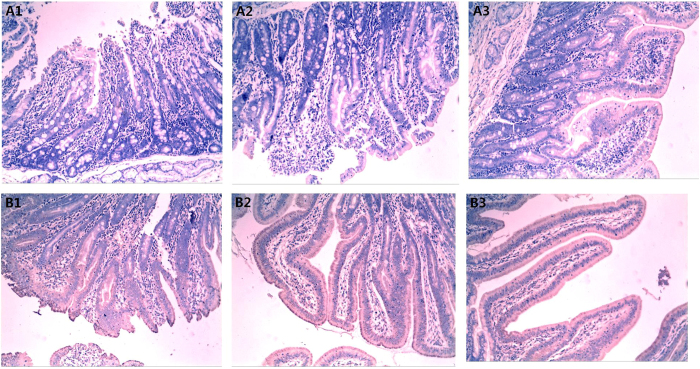
Immunohistochemical analyses in porcine duodenal tissues from *E. coli* F18-susceptible and –resistant individuals. (**A**) represents *E. coli* F18-susceptible individuals (1, 2, 3 showed three samples); (**B**) represents *E. coli* F18-resistant individuals (1, 2, 3 showed three samples, 100× magnification, scale bar = 10 μm).

**Figure 8:**
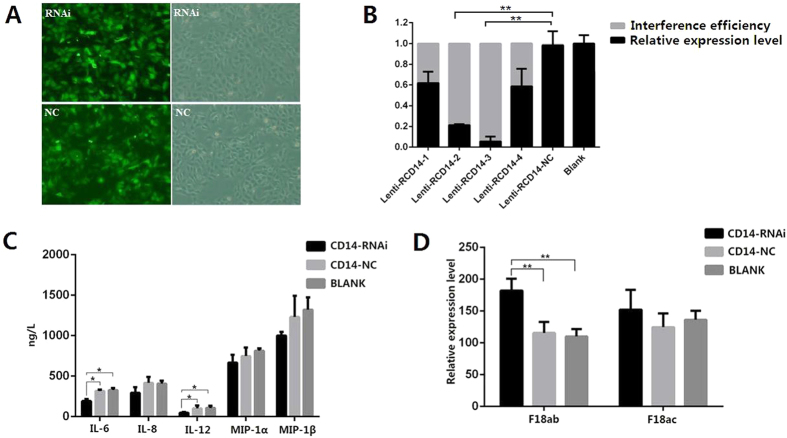
Effect of lentivirus-mediated small hairpin RNA (shRNA) silencing on *CD14* gene expression, cytokines and F18 fimbria adhesion in IPEC-J2 cells. (**A**) Green fluorescence protein (GFP) expression recorded under a fluorescence microscope (100×). (**B**) The mRNA levels of *CD14* in non-treated (Blank), constructed lentiviruses containing non-silencing small hairpin RNA (Lenti-RCD14-NC)-treated and Lenti-RCD14-treated cells determined by qRT-PCR analysis. (**C**) Detection of cytokines level in the TLR signal pathway in the supernatant of non-treated (BLANK), constructed lentiviruses containing non-shRNA-treated (CD14-NC), Lenti-RCD14-treated (CD14-RNAi) cells. (**D**) Adhesion test of the F18 fimbria to the epithelial cells of the small intestine *in vitro*.

To examine the function of *CD14*, we primarily performed the immunohistochemical analysis of *CD14* in the *E. coli* F18-resistant and -susceptible piglets. Immunohistochemical staining showed the increased expression of *CD14* gene was shown in the *E. coli* F18-resistant individuals compared to *E. coli* F18-susceptible individuals (Fig. 7). Then, we applied lentivirus-mediated RNAi to specifically suppress *CD14* in the pig epithelial cell line IPEC-J2. As shown in Fig. 8A, the ratio of cells with green fluorescence protein expression in shRNA-treated cells was more than 80%, indicating a satisfying infection. As shown in Fig. 8B, the mRNA level of *CD14* was significantly (*p* < 0.01) reduced in Lenti-RCD14-2 and Lenti-RCD14-3 treated cells, compared with non-treated and Lenti-RCD14-NC treated cells. The best knockdown efficiency of *CD14* was calculated to be 94.6% in IPEC-J2 cells, which was used for subsequent investigation. After *E. coli* F18 treatment, the levels of IL-6 and IL-12 were significantly reduced in the supernatants from CD14-RNAi treated cells (*p* < 0.01; Fig. 8C), compared with non-treated and Lenti-RCD14-NC treated cells. The adhesion ability of *E. coli* F18 to IPEC-J2 cells is shown in Fig. 8D: the number of bacteria in CD14-RNAi treated cells adhering with F18ab-expressing fimbriae was significantly (*p* < 0.01) higher than that in non-treated and Lenti-RCD14-NC treated cells.

## Discussion

To date, the molecular mechanisms of piglets’ bacterial diarrhea have been unclear, and researchers have attempted to use mice models to simulate piglet diarrhea. Zhao *et al.* reported that no experimental mice developed diarrhea in a challenge experiment by feeding high doses of *E*. *coli* O_111_ strain, and no pathogenic *E*. *coli* were detected in mice feces^26^. Liu *et al.* demonstrated the same phenomenon with *E. coli* K88^27^. Feng *et al.* infected mice via intraperitoneal injection of *E. coli* F14 or F18 strains and conducted adhesion tests on mice epithelial cells *in vitro*^28^. Although the experimental mice developed diarrhea, the mechanism of bacterial diarrhea was not caused by the adhesion of pathogenic bacteria to mice epithelial cells. Therefore, using mice as model animals to simulate piglet diarrhea is not feasible, indicating that we should conduct the challenge experiment with bacteria in piglets themselves. Recently, some studies analyzed the change in gene expression and immune factor level in pigs after *Salmonella* infection^29^,^30^. Wang *et al.* reported that Yorkshire and Laiwu black pig were normal when challenged via orally *E. coli* F18 strain^31^, because piglet feed purchased from the market contained a high concentration of antibiotics, which can kill *E. coli* F18 strain to decrease the number of bacterial infections. Through summarizing and studying previous experiences, this study seriously considered the following points. Firstly, we detected rotavirus and *E. coli* (F18, K88) in piglets’ feces before challenge experiment, excluding the possibility of the experimental piglets carrying rotavirus and *E. coli*. Secondly, this study specially prepared piglet feed without antibiotics and probiotics, avoiding an adverse impact on the experiment. Thirdly, the challenge experiment was verified and validated by *E. coli* F18 bacteria detection, bacteria counting and adhesion of small intestinal epithelial cells. The above points established the effectiveness and feasibility of our piglet diarrhea model using an artificial challenge experiment.

To date, small intestine transcriptome analyses concerning ETEC infection in swine have mainly been conducted using microarrays^9^,^10^,^32^. Compared with microarrays, RNA-Seq allows to determine the transcript abundance within a larger dynamic range of expression levels, is not limited by the available genomic sequencing information during microarray production and can provide information about new isoforms. Previous studies merely analyzed ETEC infection from the cellular level and were not detailed enough to reveal the regulatory mechanism of bacterial diarrhea in piglets. In this study, we first obtained full-sib individuals that were resistant and sensitive to *E. coli* F18. To the best of our knowledge, this is the first study in which the transcription profiles of duodenum tissues were characterized by RNA-seq in full-sib piglets resistant and sensitive to *E. coli* F18. Analyses of the transcriptomes allowed us to better understand the molecular mechanisms and regulatory pathways related to the resistance to *E. coli* F18. Using RNA-seq analysis, we identified 198 DEGs (125 were upregulated and 73 downregulated) in the resistant group compared with the sensitive group. GO function and pathway enrichment analysis revealed that the DEGs were mainly involved in immune system process belonging to the biological process category and to immune system pathways. A recent study reported the mechanisms that regulate the interaction between the immune system and the microbiota, focusing on the role of resident intestinal bacteria in the development of immune responses^33^. In view of the immune system in which upregulated DEGs were involved, we identified the TLR4-mediated Toll-like receptor signaling pathway (TLRs) as a candidate pathway. The Toll-like receptor (TLR) family recognizes conserved microbial structures, such as bacterial lipopolysaccharide and viral double-stranded RNA, and activates signaling pathways that result in immune responses against microbial infections^34^. The TLRs sense microbial populations in the intestine and initiate proinflammatory signaling pathways against invading microbial pathogens^35^.

In addition, several regulated genes identified as DEGs in the present study, such as *CD14*, *CXCL11*, are significantly involved in the TLRs pathway. CXCL11, belonging to chemokine family, is produced by a variety of cells, including leukocytes, fibroblasts and endothelial cells upon stimulation with interferons (IFNs)^36^. CXCL11 and its receptor, CXCR3, are likely to be associated with important inflammatory diseases of livestock, as well as with protective immunity to infectious diseases and tumors. Su *et al.* reported that CXCL11 was predominantly expressed in CD^+^_14_ cells of the mouse colonic mucosa lamina, producing proinflammatory effects in inflammatory bowel disease (IBD) and they interacted with a bacterial flagellum protein^37^, suggesting that the immune regulation of *CXCL11* is closely related to the expression level of *CD14*. CD14, as the adaptor molecule of TLR signaling pathway, plays an important role in bacterial infection as a high affinity receptor of lipopolysaccharide (LPS), which activated intracellular signaling pathways, ultimately leading to release cytokines^38^. Moreover, the DEGs identified here were also involved in other infectious diseases, including pathogenic *Escherichia coli* infection pathway and *Salmonella* infection pathway, in both of which the *CD14* gene plays a significant role. Systemic *CD14*-inhibition efficiently, though organ dependent, attenuated local inflammatory responses in porcine *Escherichia coli* sepsis^39^. CD14, as a key upstream innate immunity molecule, was related to the early inflammatory and hemostatic responses in a pig model of gram-negative sepsis^40^. As can be seen, *CD14* gene plays a role in immune and inflammatory regulation of porcine with *E. coli* infection. In this study, the expression of *CD14* was regulated in LPS-induced IPEC-J2 cells by qPCR and western blot, meanwhile immunohistochemical analysis showed the increased expression of *CD14* in the *E. coli* F18-resistant individuals compared to F18-susceptible. Furthermore, the knockdown of *CD14* caused significant reductions in the levels of the cytokines IL-6 and IL-12 in the IPEC-J2 cell supernatants. Meanwhile the adhesion ability of F18ab strain with IPEC-J2 cells was significantly increased (*p* < 0.01). IL-6 is a cytokine with multiple biological activities, some of which are involved in various aspects of immune and inflammatory responses^41^. Decreases expression of IL-6 in swine lymphocytes leads to continuous immunosuppression^42^. IL-12 has extensive capacity to activate cytotoxic lymphocytes, including Th1-mediated CD4+ cell differentiation, and stimulates the activation of natural killer (NK) cells and the production of INF-γ^43^; therefore, it has been considered a target gene for bioprevention. Wu *et al.*^44^ demonstrated that the higher expression of IL-6 or IL-12 contributed to improving the general disease resistance in pigs^44^. Thus, we speculated the *CD14* gene probably represents an important candidate gene related to the resistance to *E. coli* F18 in Chinese Meishan pigs.

In this study, we identified a differential response of the TLR4-mediated TLR signaling pathway and the *CD14* gene in Meishan full-sib individuals resistant and sensitive to *E. coli* F18 using RNASeq transcriptome analysis. In future studies, we will further analyze the function of TLR4 signaling pathway genes and important functional genes (e.g., *CD14*) by CRISPR /Cas9 technology, to confirm whether these genes affect the piglets’ resistance against *E. coli* F18 infection, which will provide new methods and strategies for breeding Chinese domestic pig breeds that are resistant to *E. coli* F18.

## Additional Information

**How to cite this article**: Wu, Z. *et al.* *CD14* in the TLRs signaling pathway is associated with the resistance to *E. coli* F18 in Chinese domestic weaned piglets. *Sci. Rep.* **6**, 24611; doi: 10.1038/srep24611 (2016).

## Supplementary Material

Supplementary Information
