# Supplementary material for: CD14 in the TLRs signaling pathway is associated with the resistance to E. coli F18 in Chinese domestic weaned piglets
Source: Sci Rep. 2016 Apr 21;6:24611. doi: 10.1038/srep24611 (PMC4838916; doi:10.1038/srep24611)
Supplement: Supplementary Information [file srep24611-s1.pdf]

CD14 in the TLRs signaling pathway is associated with the resistance to *E. coli* F18 in Chinese domestic weaned piglets

Zhengchang Wu[1], Ying Liu[1], Wenhua Dong[1], Guo-qiang Zhu[2], Shenglong Wu[1] & Wenbin Bao[1]

[1]Key Laboratory for Animal Genetics, Breeding, Reproduction and Molecular Design of Jiangsu Province, College of Animal Science and Technology, Yangzhou University, Yangzhou 225009, P. R. China. [2]College of Veterinary Medicine, Yangzhou University, Yangzhou, Jiangsu, P. R. China

Zhengchang Wu[1]

Email : wuzhengchang@126.com

Ying Liu[1]

Email: yddkly@163.com

Wenhua Dong[1]

Email: dingdang626@163.com

Guoqiang Zhu[2]

Email: yzgqzhu@yzu.edu.cn

Shenglong Wu[1]

Email: slwu@yzu.edu.cn

Corresponding author: Wenbin Bao

Address: Key Laboratory for Animal Genetics, Breeding, Reproduction and Molecular Design of Jiangsu Province, College of Animal Science and Technology, Yangzhou University, Yangzhou, Jiangsu 225009, P. R. China

Tel.: 86-514-87979350; Fax: 86-514-87350440;

E-mail: wbbao@yzu.edu.cn

Supplementary information

Table S1

Primers amplifying common pathogens causing diarrhea in piglets

Gene	Accession no.	Sequence（5′→3′）	annealing temperature (°C)	Length of sequence (bp)
STa	13877174	F: GCTAATGTTGGCAATTTTTATTTCTGTA<br>R: AGGATTACAACAAAGTTCACAGCAGTAA	58	190
LT	S60731	F: TGAAAAATATAACTTTCATTTTTTTTATT<br>R: TAGTTTTCCATACTGATTGCCGCAATTGA	60	1100
TGEV-MP	FJ755618	F: CAACCCTGAAACTAACGCAATTCT<br>R: GCCCATCCAGTCGCACTACTT	62	252
PEDV-NP	AF353511	F: AGGAACGTGACCTGAAAGACATCCC<br>R: CCAGGATAAGAAGGTCTAACATTG	59	540
RV-VP7	FJ807867	F: AAATCCGCAACTATACTGTGACTA<br>R: TGGCCAACTGGTTCTGTCTA	61	412

Sta represents *E. coli* strain F18; *LT* represents *E. coli* strain K88; *TGEV* represents transmissible gastroenteritis virus; *PEDV* represents porcine epidemic diarrhea virus; *RV* represents rotavirus.

Table S2

Primer sequences for differentially expressed genes identified by transcriptome analysis

Gene Symbol	GenBank accession number	Primer sequences 5′-3′	Amplicon size (bp)
CD14	AB267810	F: 5'-CCTCAGACTCCGTAATGTG-3'<br>R: 5'-CCGGGATTGTCAGATAGG-3'	180
SPP1	EF633681	F:TGCAGTGATAGCCTTCTGCC<br>R:TGTGGCGCTAGGAAAGTCTG	165
CXCL11	EU682377	F:TGTTCAAAGCGGGAAGGTGT<br>R:GGCATCTTCGTCCTTTATGTGC	153
MUC4	NM_001206344	F:CTCTTTCTGGAGCCATGAGGG<br>R:GTGAAGTCCACCGTCCTTCT	193
MMP9	NM_001038004	F:ACTTCGGAAACGCAAAAGGC<br>R:AAGAGTCTCTCGCTAGGGCA	169
ALB	XM_005666736	F:GAAGCAAACTGCACTCGTTGA<br>R:TCTCAGGGTAGTCTCCACAGC	154
GAPDH	AF017079	F:5′-ACATCATCCCTGCTTCTACTGG<br>R: 5′-CTCGGACGCCTGCTTCAC	188
ACTB	XM_003124280.3	F: TGGCGCCCAGCACGATGAAG<br>R: GATGGAGGGGCCGGACTCGT	149

The selected genes were identified by real-time PCR. The housekeeping genes, *GAPDH* and *ACTB* were used as the internal controls. The data were analyzed by the cycle threshold (C(t)) method.

Table S3

Primer sequences for genes in the Toll-like receptor signaling pathway

Gene	Sequence	Length of sequence (bp)	Accession no.
TLR4	F: 5'-CAGATAAGCGAGGCCGTCATT-3'<br>R: 5'-TTGCAGCCCACAAAAAGCA-3'	113	AB232527
CD14	F: 5'-CCTCAGACTCCGTAATGTG-3'<br>R: 5'-CCGGGATTGTCAGATAGG-3'	180	AB267810
MyD88	F: 5'-GTGCCGTCGGATGGTAGT-3'<br>R: 5'-CAGTGATGAACCGCAGGAT-3'	173	EU056736
TNF-α	F: 5'-CGACTCAGTGCCGAGATCAA-3'<br>R: 5'-CCTGCCCAGATTCAGCAAAG-3'	58	X54001
IL-1β	F: 5'-TGATTGTGGCAAAGGAGGA-3'<br>R: 5'-TTGGGTCATCATCACAGACG-3'	63	NM_001005149
IFN-α	F: 5'-CCTGGACCACAGAAGGGA-3'<br>R: 5'-TCTCATGCACCAGAGCCA-3'	92	X57191
GAPDH	F: 5'-ACATCATCCCTGCTTCTACTGG-3'<br>R: 5'-CTCGGACGCCTGCTTCAC-3'	188	AF017079
ACTB	F: TGGCGCCCAGCACGATGAAG<br>R: GATGGAGGGGCCGGACTCGT	149	XM_003124280.3

The selected genes were identified by real-time PCR. The housekeeping genes, *GAPDH* and *ACTB* were used as the internal controls. The data were analyzed by the cycle threshold (C(t)) method.

Table S4

Comparison of bacterial numbers in diarrheal and normal piglets

20 μL Coated plate		Diluted multiples				Average CFU/mL	PCR detection result (Three repetitions)		
		10^4	10^5	10^6	10^7		1	2	3
Normal group	Duodenum	200	5	NG	NG	1.0×10^8	+	+	+
	Jejunum	100	NG	NG	NG	5.0×10^7	+	+	+
	Ileum	50	NG	NG	NG	2.5×10^7	+	+	+
	Rectum	35	NG	NG	NG	1.8×10^7	+	+	+
Diarrhea group	Duodenum	>1000	>1000	>600	13	6.5×10^9	+	+	+
	Jejunum	>1000	140	26	NG	7.0×10^8	+	+	+
	Ileum	>1000	>500	150	6	7.5×10^9	+	+	+
	Rectum	>1000	>500	400	25	2.0×10^{10}	+	+	+

NG represents no growth; “+” represents the detection of bacteria.

Table S5

The data quality analysis of the investigated samples

Sample	Library Type	Reads Length (bp)	Raw Reads	Raw Data (bp)	Clean Data (bp)	Q20 (%)	Q30 (%)	GC (%)
R1	Paired-End	100	40,966,293	8,193,258,600	7,702,759,076	94.01%	84.33%	49.64%
R2	Paired-End	100	47,249,775	9,449,955,000	8,885,998,536	94.03%	84.29%	50.40%
R3	Paired-End	100	31,641,237	6,391,529,874	6,244,593,324	97.70%	93.75%	56.72%
S1	Paired-End	100	39,577,426	7,915,485,200	7,438,224,314	93.97%	84.30%	49.65%
S2	Paired-End	100	48,805,107	9,761,021,400	9,168,703,145	93.93%	84.03%	50.78%
S3	Paired-End	100	36,411,035	7,355,029,070	7,167,287,043	97.45%	93.55%	56.73%

Raw reads: the raw reads after transformation from the sequenced data by base calling.

Clean reads: the reads remaining after filtering of the raw reads.

Q20: the bases amount ratio of error rate ≤1%.

Q30: the bases amount ratio of error rate ≤0.1%.

GC: the % of G & C in total bases.

Table S6

Summary of Illumina sequencing and mapping

Sample name	R1	R2	R3	S1	S2	S3
Total reads	58147534	67048462	59597388	56098450	69093942	68312282
Total mapped	47,760,626 (82.14%)	54,611,957 (81.45%)	45,877,781 (76.98%)	45,724,539 (81.51%)	56,170,889 (81.30%)	52,624,668 (77.04%)
Multiple mapped	4,016,456 (8.41%)	4,560,337 (8.35%)	3,926,805 (8.56%)	3,810,008 (8.33%)	4,625,857 (8.24%)	4,393,851 (8.35%)
Uniquely Mapped	43,744,170 (91.59%)	50,051,620 (91.65%)	41,950,976 (91.44%)	41,914,531 (91.67%)	51,545,032 (91.76%)	48,230,817 (91.65%)

R: *E. coli F18* resistance individuals

S: *E. coli F18* susceptibility individuals

Total reads: The total amount of clean reads.

Total mapped: The total reads amount mapped to the reference genome/sequence.

Multi-mapped: The reads amount mapped to the reference genome/sequence at more than one site.

Uniq-mapped: The reads amount mapped to the reference genome/sequence at only one site.

Table S7

Percentage of reads mapped for each sample and their localization (exonic, intronic or intergenic) with reference to the pig reference genome sequence (Sscrofa10.2 genome assembly).

Sample	Mapped Reads (%)	Exonic Reads (%)	Reads Mapped to Gene (%)	Intergenic Reads (%)
R1	82.14%	93.37%	83.97%	16.03%
R2	81.45%	95.31%	84.75%	15.25%
R3	76.98%	92.61%	85.99%	14.01%
S1	81.51%	93.49%	84.96%	15.04%
S2	81.30%	95.05%	86.08%	13.92%
S3	77.04%	90.45%	85.74%	14.26%
Average	80.07%	93.38%	85.25%	14.75%

R represents the group of *E. coli* F18 resistance; S represents the group of *E. coli* F18 susceptibility

Table S8

Differentially-expressed genes in duodenal tissues of individuals in the *E. coli F18*-resistant group and the susceptible group. Fold change means *E. coli* F18-sensitive group/*E. coli* F18-resistant group.

Up-regulated genes

Overlapping DE genes from DESeq analyses	Associated ID	log2Fold Change	*P*-value	Base Mean	-Log10(*P*-value)
ENSSSCG00000000211	*AQP5*	-1.24	3.46E-02	2684	1.46
ENSSSCG00000000637	*novel gene*	-1.32	1.71E-02	515	1.77
ENSSSCG00000000647	*OLR1*	-1.32	2.03E-02	195	1.69
ENSSSCG00000000688	*LAG3*	-1.34	3.19E-02	65	1.50
ENSSSCG00000000932	*NTS*	-3.06	1.31E-06	135	5.88
ENSSSCG00000001484	*novel gene*	-1.57	7.88E-03	124	2.10
ENSSSCG00000001724	*MEP1A*	-3.06	6.84E-07	1836	6.17
ENSSSCG00000001979	*novel gene*	-1.67	3.48E-02	12	1.46
ENSSSCG00000002262	*SV2B*	-2.07	1.03E-02	15	1.99
ENSSSCG00000002821	*CCL22*	-1.52	1.08E-02	95	1.97
ENSSSCG00000002980	*novel gene*	-2.55	1.38E-02	7	1.86
ENSSSCG00000003048	*CXCL17*	-1.47	1.17E-02	820	1.93
ENSSSCG00000003192	*IL4I1*	-1.56	8.04E-03	147	2.09
ENSSSCG00000003331	*TNFRSF4*	-1.63	6.53E-03	99	2.19
ENSSSCG00000003437	*TNFRSF8*	-1.86	7.63E-03	26	2.12
ENSSSCG00000003622	*CSMD2*	-2.32	4.33E-04	47	3.36
ENSSSCG00000003832	*TACSTD2*	-3.04	8.59E-04	12	3.07
ENSSSCG00000004001	*A1BG*	-4.35	1.42E-02	3	1.85
ENSSSCG00000004158	*IL22RA2*	-2.25	7.42E-03	12	2.13
ENSSSCG00000004194	*novel gene*	-1.87	1.17E-03	444	2.93
ENSSSCG00000005222	*SLC1A1*	-1.50	9.62E-03	2170	2.02
ENSSSCG00000005269	*TRPM6*	-1.76	3.98E-03	130	2.40

ENSSSCG00000005474	*novel gene*	-3.14	3.96E-02	3	1.40
ENSSSCG00000006043	*DCSTAMP*	-4.32	1.44E-02	3	1.84
ENSSSCG00000006073	*OSR2*	-2.93	2.89E-05	45	4.54
ENSSSCG00000006245	*SDR16C5*	-2.44	2.07E-04	58	3.68
ENSSSCG00000006450	*PCD1E*	-1.25	4.54E-02	69	1.34
ENSSSCG00000006472	*CRABP2*	-2.11	1.63E-03	32	2.79
ENSSSCG00000006759	*OLFML3*	-1.32	1.99E-02	1339	1.70
ENSSSCG00000006792	*PGB*	-3.42	8.21E-08	164	7.09
ENSSSCG00000007007	*IDO1*	-1.29	1.95E-02	152	1.71
ENSSSCG00000007084	*BFSP1*	-2.71	9.32E-04	17	3.03
ENSSSCG00000007385	*KCNS1*	-1.70	1.26E-02	19	1.90
ENSSSCG00000007387	*PI3*	-2.73	1.02E-06	855	5.99
ENSSSCG00000007405	*WFDC2*	-2.06	6.17E-04	120	3.21
ENSSSCG00000007436	*MMP9*	-1.42	1.96E-02	129	1.71
ENSSSCG00000008228	*GNLY*	-1.48	1.01E-02	549	2.00
ENSSSCG00000008319	*CD207*	-2.23	2.70E-02	7	1.57
ENSSSCG00000008648	*IRG6*	-1.52	1.16E-02	2156	1.93
ENSSSCG00000008948	*ALB*	-2.71	1.46E-03	12	2.83
ENSSSCG00000008957	*AMCF-II*	-2.43	1.16E-05	789	4.94
ENSSSCG00000008963	*AREG*	-1.49	8.59E-03	384	2.07
ENSSSCG00000008978	*CXCL11*	-2.28	1.65E-04	141	3.78
ENSSSCG00000009004	*SFRP2*	-1.21	3.91E-02	123	1.41
ENSSSCG00000009216	*SPP1*	-1.07	4.69E-02	238	1.33
ENSSSCG00000009240	*PLAC8*	-1.34	2.28E-02	2023	1.64
ENSSSCG00000009413	*CPB2*	-1.62	7.40E-03	82	2.13
ENSSSCG00000009469	*novel gene*	-2.65	2.18E-03	11	2.66
ENSSSCG00000009558	*F10*	-1.50	1.30E-02	112	1.88
ENSSSCG00000009856	*NOS1*	-1.33	3.83E-02	29	1.42
ENSSSCG00000010190	*ACTA1*	-2.11	7.01E-03	18	2.15
ENSSSCG00000010369	*NPY4R*	-1.68	2.15E-02	23	1.67
ENSSSCG00000010432	*novel gene*	-1.22	3.92E-02	2089	1.41
ENSSSCG00000010437	*PAPSS2*	-2.17	2.39E-04	2691	3.62
ENSSSCG00000010453	*IFIT1*	-1.58	7.86E-03	4993	2.10
ENSSSCG00000010546	*CYP2C33*	-3.50	6.14E-03	5	2.21
ENSSSCG00000011147	*novel gene*	-1.52	8.93E-03	2251	2.05
ENSSSCG00000011148	*novel gene*	-1.63	2.81E-02	22	1.55
ENSSSCG00000011361	*SLC26A6*	-2.66	1.02E-05	505	4.99
ENSSSCG00000011723	*MME*	-1.80	2.17E-03	5466	2.66
ENSSSCG00000011736	*SLITRK3*	-2.78	1.97E-04	25	3.71
ENSSSCG00000011850	*MUC4*	-3.02	1.88E-06	64	5.73
ENSSSCG00000012277	*TIMP1*	-1.13	3.93E-02	624	1.41
ENSSSCG00000012485	*NOX1*	-1.04	4.77E-02	135	1.32
ENSSSCG00000013599	*ANGPTL4*	-1.34	1.62E-02	953	1.79
ENSSSCG00000013901	*IFI30*	-1.10	4.51E-02	2215	1.35

ENSSSCG00000014048	*HK3*	-1.23	2.77E-02	481	1.56
ENSSSCG00000014111	*BHMT2*	-4.70	4.96E-03	4	2.30
ENSSSCG00000014130	*novel gene*	-1.73	1.14E-02	34	1.94
ENSSSCG00000014186	*novel gene*	-4.12	6.53E-08	38	7.19
ENSSSCG00000014369	*CD14*	-1.22	3.64E-02	220	1.44
ENSSSCG00000014725	*HBB*	-1.10	4.53E-02	632	1.34
ENSSSCG00000014982	*MMP7*	-3.94	5.29E-09	94	8.28
ENSSSCG00000015476	*CHI3L1*	-1.55	6.40E-03	257	2.19
ENSSSCG00000015895	*GCG*	-2.22	1.26E-04	225	3.90
ENSSSCG00000016135	*CPO*	-5.98	6.45E-06	10	5.19
ENSSSCG00000016348	*novel gene*	-1.57	7.89E-03	40	2.10
ENSSSCG00000016609	*SLC13A1*	-4.17	8.70E-11	471	10.06
ENSSSCG00000016867	*CCDC152*	-1.65	3.96E-02	14	1.40
ENSSSCG00000016868	*ANXA2R*	-2.07	2.96E-03	25	2.53
ENSSSCG00000016941	*RNF180*	-2.28	2.48E-02	8	1.61
ENSSSCG00000017163	*ENPP7*	-2.24	2.13E-04	2253	3.67
ENSSSCG00000017249	*SSTR2*	-1.44	1.84E-02	112	1.73
ENSSSCG00000017466	*CCR7*	-1.18	4.60E-02	126	1.34
ENSSSCG00000017798	*TMIGD1*	-4.55	2.97E-11	158	10.53
ENSSSCG00000021208	*SEPP1*	-1.28	2.51E-02	21183	1.60
ENSSSCG00000022094	*CA7*	-4.32	1.35E-05	13	4.87
ENSSSCG00000022549	*novel gene*	-8.07	1.59E-13	40	12.80
ENSSSCG00000022913	*SLPI*	-1.26	2.88E-02	115	1.54
ENSSSCG00000023566	*novel gene*	-1.62	2.39E-02	26	1.62
ENSSSCG00000023612	*novel gene*	-1.41	3.21E-02	44	1.49
ENSSSCG00000023740	*novel gene*	-1.67	3.82E-03	341	2.42
ENSSSCG00000023756	*novel gene*	-4.16	1.71E-08	43	7.77
ENSSSCG00000023762	*TRPM2*	-1.56	1.29E-02	56	1.89
ENSSSCG00000024634	*C10orf99*	-1.58	1.17E-02	41	1.93
ENSSSCG00000025020	*PCSK9*	-2.25	1.20E-04	207	3.92
ENSSSCG00000025795	*CSF3R*	-1.39	1.80E-02	127	1.75
ENSSSCG00000025992	*novel gene*	-1.97	6.94E-04	487	3.16
ENSSSCG00000026043	*TGM3*	-1.47	1.28E-02	93	1.89
ENSSSCG00000026131	*novel gene*	-1.66	4.74E-02	14	1.32
ENSSSCG00000026286	*novel gene*	-2.14	2.98E-03	27	2.53
ENSSSCG00000026333	*GTSF1*	-5.25	1.56E-06	13	5.81
ENSSSCG00000026336	*novel gene*	-4.25	1.84E-02	3	1.74
ENSSSCG00000026594	*SULT6B1*	-7.81	2.61E-24	1023	23.58
ENSSSCG00000026674	*novel gene*	-1.38	4.72E-02	21	1.33
ENSSSCG00000027322	*PGLYRP3*	-1.67	3.32E-02	16	1.48
ENSSSCG00000027867	*novel gene*	-1.20	3.76E-02	202	1.42
ENSSSCG00000028127	*novel gene*	-1.77	8.95E-03	40	2.05
ENSSSCG00000028173	*TRIM31*	-1.57	2.53E-03	484	2.60
ENSSSCG00000028309	*novel gene*	-1.64	4.32E-03	719	2.36

ENSSSCG00000028642	*novel gene*	-2.19	2.23E-04	189	3.65
ENSSSCG00000028817	*novel gene*	-2.01	1.02E-02	14	1.99
ENSSSCG00000028839	*novel gene*	-3.10	8.05E-03	6	2.09
ENSSSCG00000028982	*AKR*	-3.97	3.99E-02	2	1.40
ENSSSCG00000029416	*novel gene*	-2.76	1.97E-03	11	2.70
ENSSSCG00000029714	*BPIFB2*	-2.42	2.83E-02	5	1.55
ENSSSCG00000030425	*novel gene*	-6.42	1.39E-14	69	13.86
ENSSSCG00000030585	*HOXC6*	-1.52	2.20E-02	31	1.66
ENSSSCG00000030706	*FAM43B*	-1.87	7.39E-03	27	2.13
ENSSSCG00000030888	*FADD*	-5.61	1.01E-10	37	10.00
ENSSSCG00000000239	*novel gene*	-Inf	7.58E-04	4	3.12
ENSSSCG00000010899	*novel gene*	-Inf	3.23E-02	2	1.49
ENSSSCG00000011799	*AHSG*	-Inf	9.39E-03	3	2.03
ENSSSCG00000012871	*FGF19*	-Inf	3.61E-03	3	2.44
ENSSSCG00000027874	*novel gene*	-Inf	1.70E-03	3	2.77

Down-regulated genes

Overlapping DE genes from DESeq analyses	Associated ID	log2Fold Change	*P*-value	Base Mean	-Log10(*P*-value)
ENSSSCG00000000046	*CYP2D25*	1.28	1.72E-02	243	1.76
ENSSSCG00000000667	*AICDA*	1.48	3.16E-02	22	1.50
ENSSSCG00000001749	*novel gene*	1.61	4.45E-02	22	1.35
ENSSSCG00000003006	*CYP2B22*	1.24	2.21E-02	480	1.66
ENSSSCG00000003247	*novel gene*	1.51	7.25E-03	215	2.14
ENSSSCG00000003336	*TTLL10*	4.14	1.84E-02	3	1.74
ENSSSCG00000003368	*RNF207*	1.72	4.83E-03	61	2.32
ENSSSCG00000003835	*C8A*	1.37	1.34E-02	1167	1.87
ENSSSCG00000004699	*ELL3*	3.06	1.58E-06	76	5.80
ENSSSCG00000005287	*PSAT1*	1.14	4.08E-02	239	1.39
ENSSSCG00000005315	*CA9*	1.51	5.26E-03	1874	2.28
ENSSSCG00000005360	*IGFBPL1*	2.43	3.14E-03	13	2.50
ENSSSCG00000005843	*LCN15*	2.31	4.12E-04	62	3.38
ENSSSCG00000005862	*novel gene*	1.15	2.92E-02	308	1.53
ENSSSCG00000006286	*SELE*	1.39	4.02E-02	68	1.40
ENSSSCG00000007549	*CYP2W1*	1.41	1.12E-02	131	1.95
ENSSSCG00000007691	*ZP3*	2.36	3.58E-02	5	1.45
ENSSSCG00000007807	*CD19*	1.34	2.02E-02	90	1.70
ENSSSCG00000007861	*novel gene*	4.60	1.91E-07	21	6.72
ENSSSCG00000008142	*C2orf40*	1.41	1.80E-02	65	1.75
ENSSSCG00000008954	*novel gene*	2.97	2.40E-02	4	1.62
ENSSSCG00000009789	*GPR81*	2.35	4.16E-02	6	1.38
ENSSSCG00000010075	*VPREB3*	1.70	8.81E-03	34	2.06
ENSSSCG00000010210	*SLC16A9*	1.80	1.70E-03	167	2.77
ENSSSCG00000011800	*FETUB*	2.22	1.09E-03	33	2.96

ENSSSCG00000012049	*KCNE1*	2.29	5.45E-05	713	4.26
ENSSSCG00000012178	*novel gene*	1.66	2.58E-03	1034	2.59
ENSSSCG00000014575	*SCUBE2*	1.48	7.15E-03	273	2.15
ENSSSCG00000014876	*MYO7A*	1.29	2.08E-02	362	1.68
ENSSSCG00000015630	*novel gene*	1.21	2.63E-02	266	1.58
ENSSSCG00000016040	*novel gene*	3.57	1.17E-02	4	1.93
ENSSSCG00000016254	*CCL20*	1.39	2.57E-02	600	1.59
ENSSSCG00000016829	*AGXT2*	2.07	1.12E-03	58	2.95
ENSSSCG00000017037	*FABP6*	3.70	2.56E-08	74	7.59
ENSSSCG00000017220	*OTOP3*	1.12	3.77E-02	758	1.42
ENSSSCG00000017495	*novel gene*	1.18	3.05E-02	303	1.52
ENSSSCG00000021073	*C1orf222*	1.57	4.04E-02	15	1.39
ENSSSCG00000021357	*novel gene*	2.72	1.95E-02	5	1.71
ENSSSCG00000021812	*MS4A1*	2.37	5.83E-05	149	4.23
ENSSSCG00000021903	*novel gene*	2.61	1.05E-04	66	3.98
ENSSSCG00000022737	*novel gene*	2.98	5.64E-05	24	4.25
ENSSSCG00000022894	*GZMH*	2.58	3.47E-03	11	2.46
ENSSSCG00000023478	*novel gene*	1.33	1.63E-02	489	1.79
ENSSSCG00000023508	*BCMO1*	1.17	3.25E-02	211	1.49
ENSSSCG00000023537	*SYT8*	1.34	3.41E-02	32	1.47
ENSSSCG00000024208	*F12*	1.32	1.93E-02	200	1.71
ENSSSCG00000024422	*novel gene*	1.18	4.01E-02	132	1.40
ENSSSCG00000024627	*novel gene*	2.15	1.31E-03	31	2.88
ENSSSCG00000024911	*novel gene*	1.30	2.71E-02	58	1.57
ENSSSCG00000024943	*EHD3*	1.39	2.19E-02	58	1.66
ENSSSCG00000025723	*novel gene*	3.85	1.13E-05	16	4.95
ENSSSCG00000025853	*MYL6*	2.03	1.87E-03	47	2.73
ENSSSCG00000026430	*novel gene*	1.28	1.83E-02	1406	1.74
ENSSSCG00000026572	*HIST1H2BK*	2.12	3.66E-02	7	1.44
ENSSSCG00000027093	*FOLH1*	1.38	1.42E-02	710	1.85
ENSSSCG00000027157	*novel gene*	2.79	6.85E-05	33	4.16
ENSSSCG00000028106	*novel gene*	1.20	4.41E-02	61	1.36
ENSSSCG00000028674	*CR2*	2.97	5.03E-07	342	6.30
ENSSSCG00000028881	*CNIH3*	1.30	3.97E-02	51	1.40
ENSSSCG00000028936	*novel gene*	1.22	3.37E-02	124	1.47
ENSSSCG00000029588	*novel gene*	3.52	1.20E-02	4	1.92
ENSSSCG00000029783	*MKX*	2.54	3.71E-02	5	1.43
ENSSSCG00000029864	*EIF1AX*	2.72	3.01E-06	597	5.52
ENSSSCG00000029902	*C11orf80*	2.35	2.32E-04	76	3.63
ENSSSCG00000030074	*UGT2B31*	1.66	8.05E-03	34	2.09
ENSSSCG00000030137	*C11orf35*	1.21	4.25E-02	46	1.37
ENSSSCG00000030246	*FCRLA*	1.88	4.19E-03	32	2.38
ENSSSCG00000030264	*novel gene*	1.45	4.10E-02	20	1.39
ENSSSCG00000030511	*novel gene*	1.90	1.61E-02	13	1.79

ENSSSCG00000004041	*novel gene*	Inf	1.35E-04	5	3.87
ENSSSCG00000005752	*BLG*	Inf	2.58E-03	3	2.59
ENSSSCG00000014916	*novel gene*	Inf	9.16E-05	6	4.04
ENSSSCG00000030371	*novel gene*	Inf	4.83E-02	2	1.32

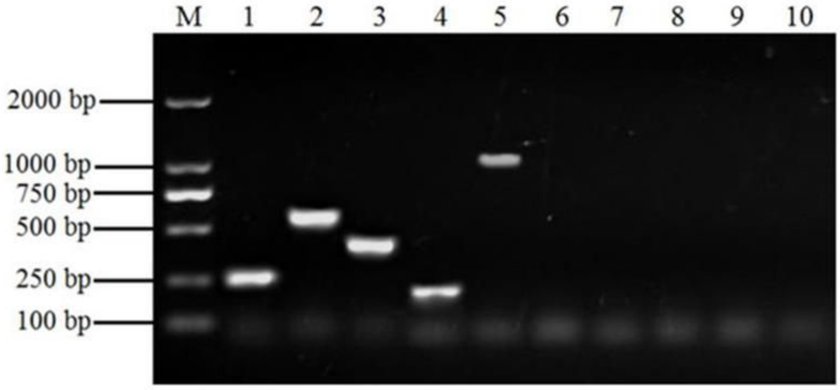

Fig. S1. PCR detection of enterotoxin genes before bacterial challenge. Lane M represents DL2000 marker; Lane 1 represents TGEV-MP; Lane 2 represents PEDV-NP; Lane 3 represents RV-VP7; Lane 4 represents *E. coli* F18; Lane 5 represents *E. coli* K88; Lane 6-10 represent PCR detection of enterotoxin genes from piglets' feces.

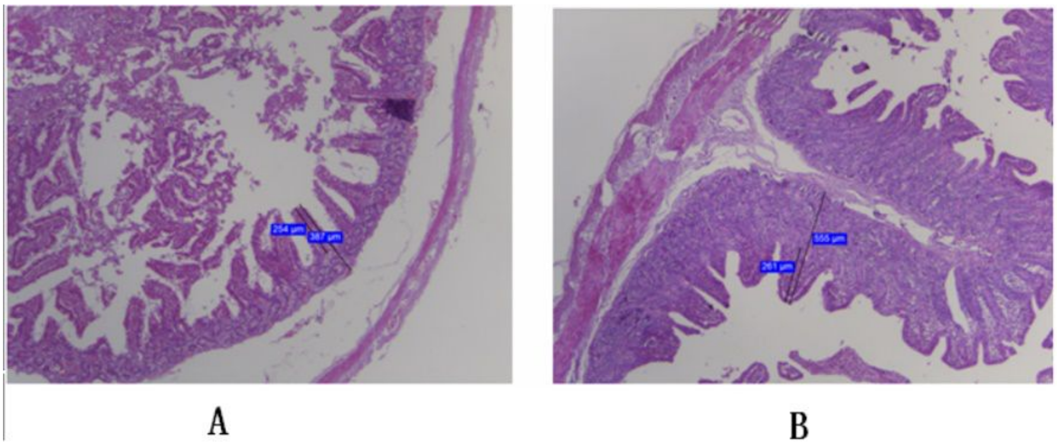

Fig. S2. The Changes in intestine tissue of a severe diarrheal pig and a normal pig after bacterial challenge (a light microscope at 40× magnification). Note: A represents pathological tissue, B represents normal tissue.

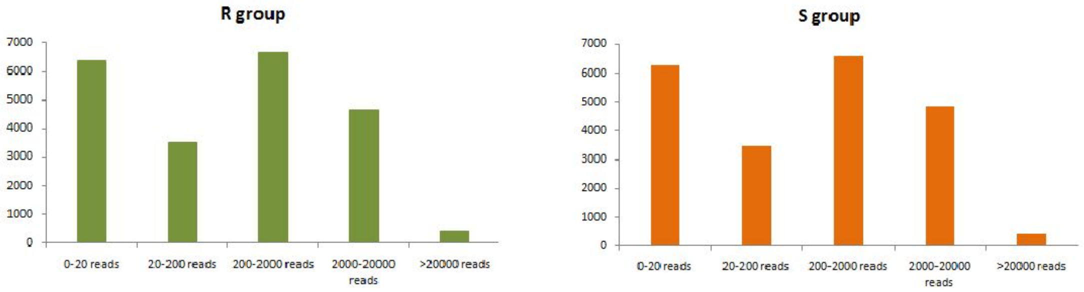

Fig. S3. Profile of gene expression distribution in groups susceptible (S) and resistant groups (R) to *E. coli* F18.

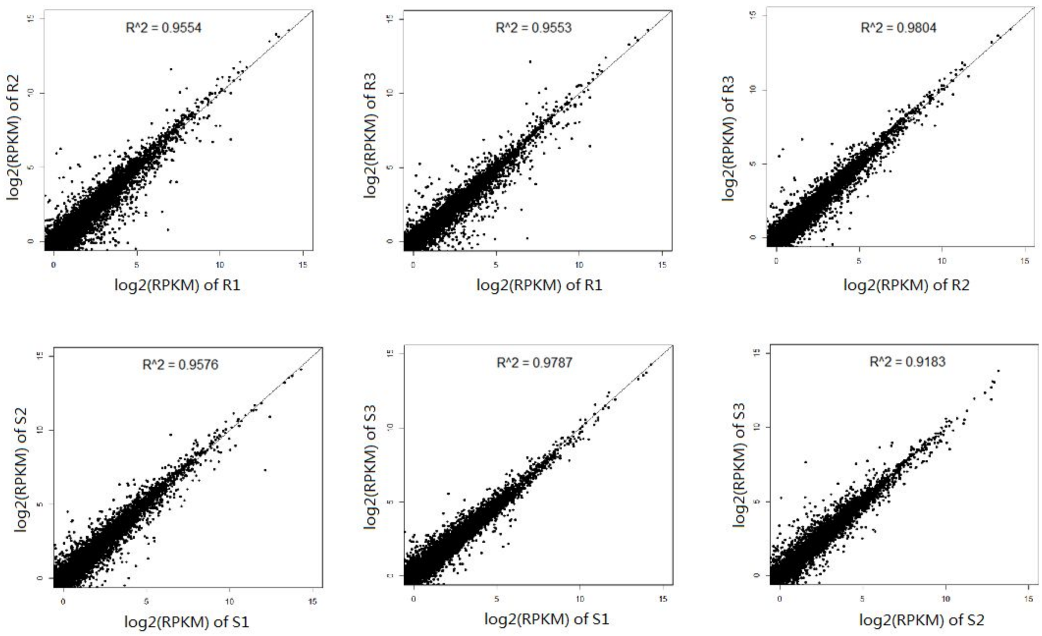

Fig. S4. Correlations between expression values of genes for two groups. X-axis and y-axis shows the log2 (FPKM S1_S2_S3) and log2 (FPKM R1_R2_R3), respectively.

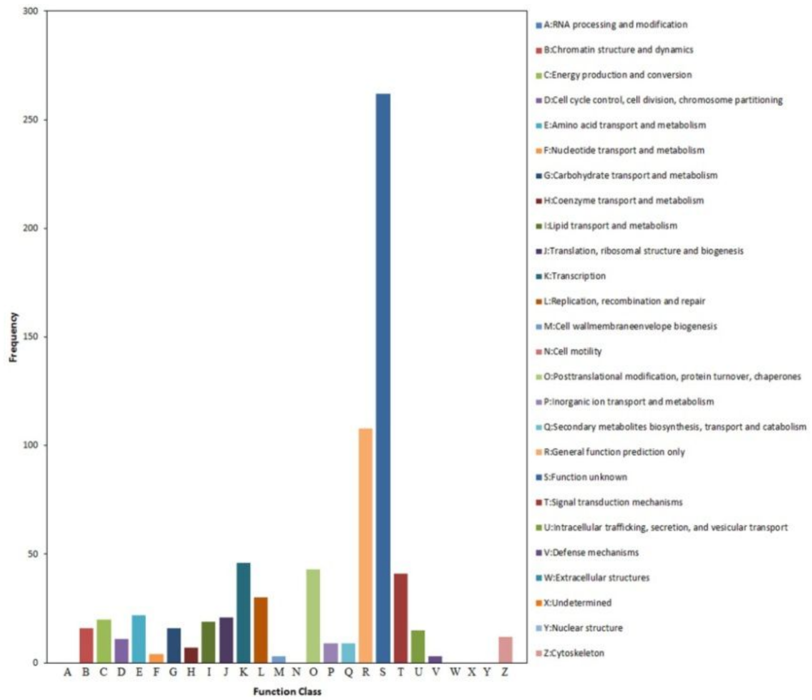

Fig. S5. EggNOG functional classification of assembled transcripts. 717 unigenes were assigned to 20 categories in the EggNOG classification. On the y-axis, ‘Frequency’ indicates the number of genes in a specific function cluster. The right legend shows a description of the 20 functional categories.

Alternative Splicing	Graph	R & S	R	S
Alternative 5' donor sites	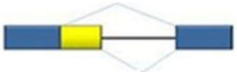	274	888	913
Alternative 3' acceptor sites	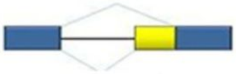	624	2117	2140
Intron retention	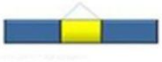	590	1819	1905
Exon skipping	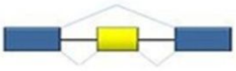	725	1509	1468

Fig. S6. Statistics of main alternative splicing events. The first column shows the types of alternative transcript events, including 5′splicing site (A5SS), alternative 3′ splicing site (A3SS), Exon skipping (ES) and intron retained (IR); the second column shows the splicing graphs and the third to fifth columns shows the number of AS (Alternative Splice) events in *E. coli* F18 susceptible (S) and *E. coli* F18 resistant (R) combined group, S group and R group, respectively.

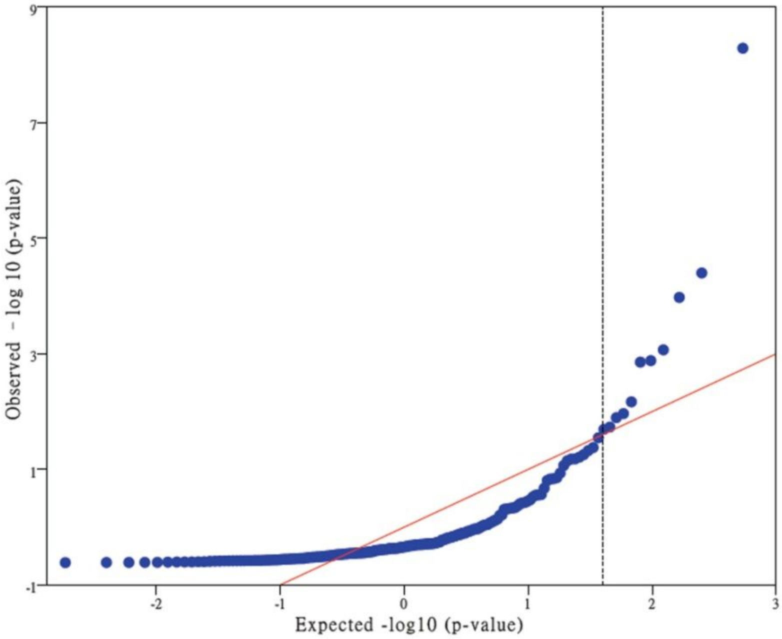

Fig. S7. Q-Q plot representing the distribution of the p-value. The red line represents the expected distribution of the p-value, while the blue trend represents the observed distribution. X-axis values are expected -log10 (p-value) and y-axis values are the observed –log 10 (p-value). The selected cut-off is represented by a discontinuous line (-log10 (p-value)>1.7).

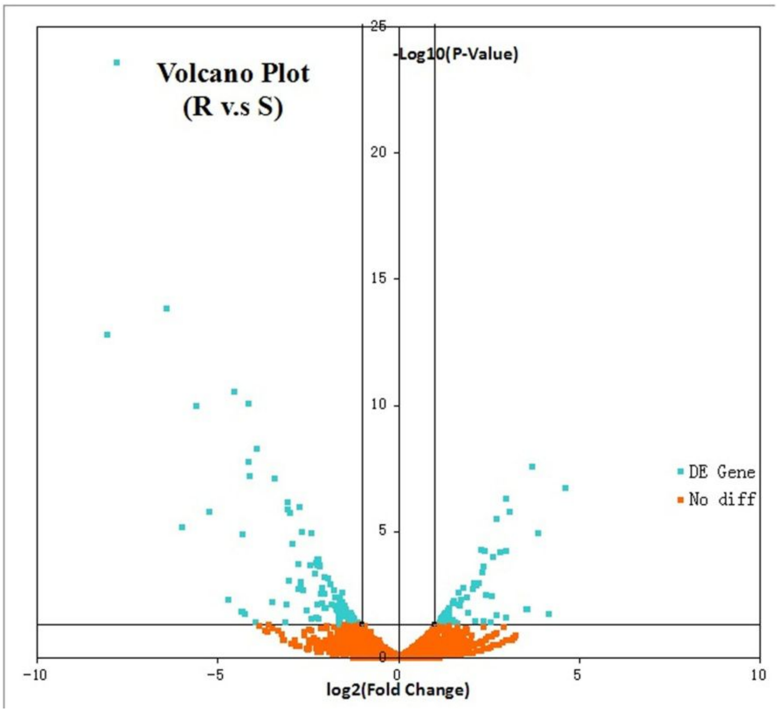

Fig. S8. Volcano plot displaying differentially expressed genes between *E. coli* F18 resistant and susceptible groups. The *y*-axis corresponds to the mean expression value of the $\log_{10}$ (p-value) and the *x*-axis values displays the $\log_2$ (fold-change) value. The blue dots represent the significantly differentially expressed transcripts ($p < 0.05$, ｜fold change｜≥2) between the *E. coli* F18 susceptible (S) and *E. coli* F18 resistant (R) groups. The left region of *y*-axis represents up-regulated genes in R group; the right region of *y*-axis represents down-regulated genes in R group.

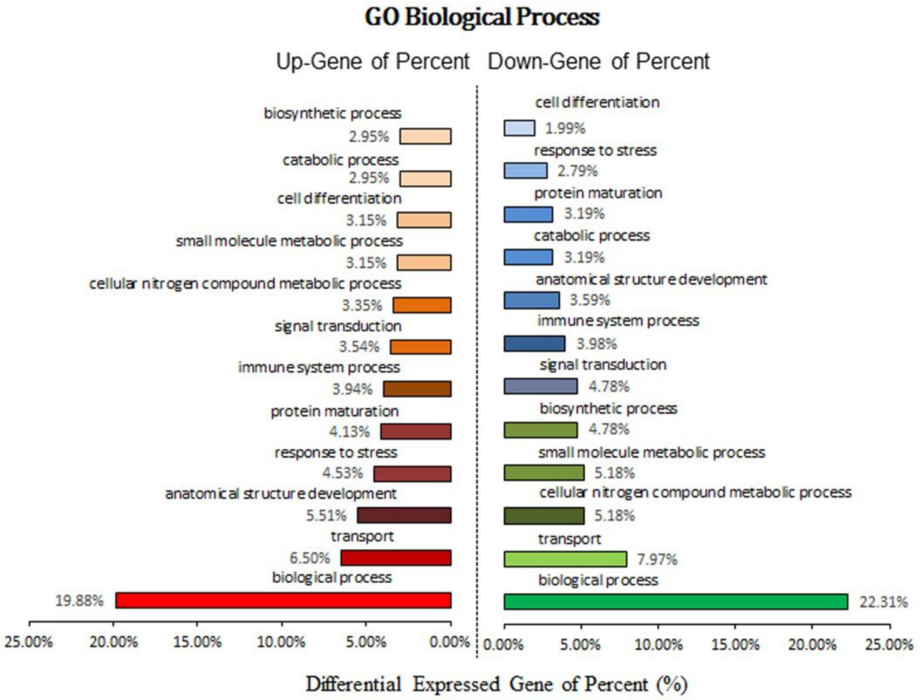

Fig. S9. GO categories based on biological processes for differentially expressed genes. The significant GO categories for differentially expressed genes; the DE genes mainly clustered into 12 functional groups. The left region represents significant GO categories for up-regulated genes in R group; the right region represents significant GO categories down-regulated genes in R group.

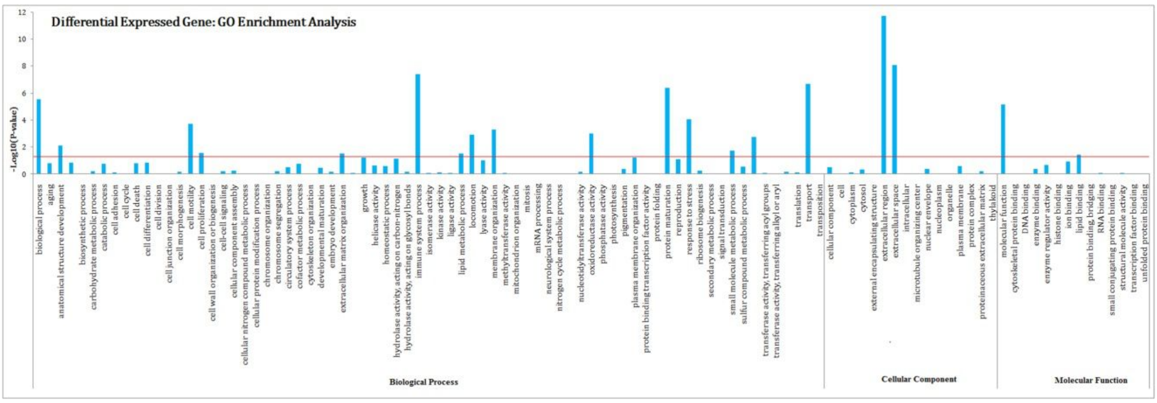

Fig. S10. Functional categorization of differentially expressed genes based on known genes in the Uniprot database. The x-axis shows the 2nd level Gene Ontology terms from biological process, cellular component and molecular function ; y-axis shows the degree of enrichment according to the $\log_{10}$ (p-value) value.
